# Active-flow systems enhance respirator fit success in children: a comparative study with standard disposable models

**DOI:** 10.3389/fpubh.2026.1851331

**Published:** 2026-10-14

**Authors:** Sittiporn Ruengdit, Kampanat Wangsan, Wuttipat Kiratipaisarl, Krongporn Ongprasert

**Affiliations:** 1 Department of Forensic Medicine, Faculty of Medicine, Chiang Mai University, Chiang Mai, Thailand; 2 Department of Community Medicine, Faculty of Medicine, Chiang Mai University, Chiang Mai, Thailand; 3 Environmental and Occupational Medicine Excellence Center (EnOMEC), Faculty of Medicine, Chiang Mai University, Chiang Mai, Thailand

**Keywords:** active-flow respirators, air pollution, children, facial anthropometry, quantitative fit testing, respiratory protection devices

## Abstract

**Introduction:**

Children face disproportionate air pollution health risks due to their physiology and behavior. Respirator efficacy data in children remain limited. This study compared standard respirators with a modified active-flow system providing positive pressure.

**Methods:**

Quantitative fit testing was performed on 82 children aged 5–10 years in Chiang Mai, Thailand, using the TSI Portacount Pro Plus 8,038. Success was defined by an overall mean fit factor of 100 or greater. The evaluation compared a horizontal triple-fold KN95-equivalent respirator with an active-flow model by connecting the mask to a system that provides a continuous supply of purified air from a portable HEPA-filtered unit delivering 66.5 liters per minute. Eight facial dimensions were measured via 2D photogrammetry to identify anthropometric correlations with performance. Fit testing incorporated four standardized exercises, namely bending, talking, side-to-side head turning, and up-and-down head movement, following a modified OSHA quantitative fit-testing protocol.

**Results:**

Standard filtering facepiece respirators (FFRs) achieved a pass rate of only 3.7%. The active-flow system significantly increased the pass rate to 23.2%, with an odds ratio of 17.0, and increased the overall median fit factor from 9.5 to 39.5. Fit success was positively associated with bizygomatic breadth (aOR 1.31) and menton-sellion length (aOR 1.11), whereas a wider interpupillary distance was associated with lower odds of passing (aOR 0.67). Among the tested exercises, up-and-down head movements resulted in the lowest fit improvements. The participants reported significantly easier breathing under the active-flow condition (86.6%) than under the standard respirator (37.8%), although inconvenience was reported more frequently with the active-flow model (76.8%) than with the standard model (35.4%).

**Conclusion:**

These findings suggest that FFRs commonly available in consumer markets may offer only limited protection for children against environmental pollutants. Active-flow systems appeared to improve both efficacy and respiratory comfort, though overall protection levels observed in this evaluation remained below thresholds generally considered protective. Given the scope of this evaluation, further research is needed to confirm these findings across broader conditions and populations, with particular attention to improving nasal seal integrity and developing portable, comfortable-to-use units with adjustable flow rates suited to real-world activity levels.

## 1 Introduction

As global air pollution levels increase, children experience disproportionately high exposure to fine particulate matter and more severe health impacts than adults do because of their unique physiological and behavioral characteristics (1). Physiologically, children possess immature detoxification mechanisms and higher minute ventilation rates because they inhale a larger volume of air per unit of body weight. Furthermore, their shorter stature positions their breathing zone closer to the ground where pollutants frequently accumulate. Behaviorally, children spend more time outdoors and lack the autonomy to mitigate their environmental exposure (1–3). Beyond immediate clinical complications such as eczema or respiratory irritation, early-life exposure is associated with irreversible or chronic health outcomes, including impaired neurodevelopment, an increased risk of noncommunicable diseases, and a heightened susceptibility to various cancers in adulthood (4–6).

Various effective individual interventions aimed at reducing outdoor pollution exposure have been recommended, although each approach presents unique implementation barriers (7). Behavioral strategies such as staying indoors and reducing physical activity avoid direct financial costs, yet these measures impose substantial burdens by limiting opportunities for exercise and experiential learning. Conversely, technical solutions such as air filtration introduce significant financial hurdles that remain inaccessible to a large portion of the population (7). Consequently, respirators often emerge as a more feasible and accessible alternative for individual protection. Evidence from northern Thailand supports this finding, as 59% of urban residents expressed a willingness to pay for filtering facepiece respirators (FFRs) compared with only 10% for air purifiers. Furthermore, rural participants in the same region showed nearly zero willingness to invest in air purification technology (8). These trends align with caregiver behavioral intentions in Hong Kong, where ensuring that a child wears a respirator is preferred over restricted indoor confinement during peak pollution periods (9). Research in Vietnam further indicates that the high local availability of respirators significantly influences their selection as the primary protective strategy for children (10).

Although FFRs are a common choice for parents, a significant risk exists regarding the misunderstanding of mask effectiveness. This misconception can create a false sense of security and encourage individuals to spend more time outdoors in polluted environments under the mistaken belief that they are adequately protected (11). A primary concern remains the lack of efficacy data specifically for children. Furthermore, adult studies on common FFRs have demonstrated poor results in terms of efficiency. A recent evaluation of the fitted filtration efficiency (FFE) of ear-loop FFRs showed that the mean FFE for KN95, surgical, and KF94 models failed to reach standard efficiency requirements (12). Additional research involving ninety quantitative face-fit tests on nine different FFR models reported that only two tests achieved the required pass rate, which represents a success rate of only 2.2% (13). The development of effective respirators for children is further complicated by the dynamic nature of a child’s face, which is undergoing continuous growth (14). This growth pattern differs by gender, with facial dimorphism emerging well before puberty (15). Fit-test outcomes and facial dimensions were therefore examined separately by gender throughout this study, since even minor facial shifts can affect the seal, a critical indicator of respirator efficacy, and thus alter quantitative fit factors (16). Although modern respirators for children may incorporate silicone edges to improve seal integrity across diverse facial structures (17, 18), such designs generally command a higher price than standard disposable masks, and cost is likely to remain one of the barriers influencing purchasing decisions for protective equipment against air pollution in Thailand (8). Beyond cost, other studies have reported additional barriers to respirator adoption, including heat accumulation (19) and discomfort from reduced vocal clarity (20), both of which merit consideration in future development.

In the context of declining global air quality, caregivers commonly rely on FFRs despite a notable lack of evidence regarding their clinical performance in children. Therefore, this study aims to evaluate the efficacy of locally accessible FFRs through standard fit testing while exploring the specific facial dimensions that may influence mask performance. Furthermore, this research investigates a modified active-flow respirator concept, which is designed to deliver a continuous supply of filtered air through a small delivery tube (21). By maintaining elevated internal pressure to create a positive-pressure microenvironment, this system attempts to address the challenges posed by diverse facial structures in children and might assist in minimizing inward contaminant leakage. The findings of this study are offered as preliminary data to support public health awareness and healthcare policy while potentially aiding the future development of respiratory protection designed for children.

## 2 Method

### 2.1 Participants and data collection

The study population consisted of children aged 5–10 years from a primary school in Chiang Mai, Thailand. The research complied with the Declaration of Helsinki and received approval from the Research Ethics Committee of the Faculty of Medicine at Chiang Mai University (Study Code COM-2567-0169). Parents or guardians provided written informed consent, and written assent was collected from participating children older than 7 years. Participants were excluded if they presented with symptoms of an upper respiratory tract illness (e.g., nasal congestion, rhinorrhea, or cough), facial skin conditions such as abrasions, irritation, or active dermatitis, or a poorly fitting bandage or dressing over the nose or cheeks, any of which could affect comfortable mask wearing on the testing days. Sample size calculations via G*Power software revealed that 68 participants were required to detect a moderate effect size (Cohen’s d = 0.35) with a two-sided significance level (alpha) of 0.05 and 80% statistical power. To accommodate potential missing data, an anticipated dropout rate of 15% was incorporated. The final targeted sample size was 80 participants, 82 of whom were ultimately recruited. Data collection occurred at the schools. After receiving a standardized protocol explanation in groups of five, the children were evaluated individually. Researchers measured height and weight and captured two photographs for facial measurements. The children then underwent quantitative mask fit testing twice. They were tested first with standard FFRs and subsequently with active-flow FFRs. Finally, subjective comfort was assessed through interviews conducted by a single investigator throughout the duration of the study. The participants indicated their perceived comfort for both interventions by pointing to a facial scale featuring unhappy, neutral, and happy expressions, which corresponded to scores of 1, 2, and 3, respectively.

### 2.2 Respirator model

The standard FFR used in this study was a commercially available four-layer respirator incorporating double melt-blown filter media of medical-grade material, as shown in Figure 1. The device is registered as a medical device and is classified as a KN95-equivalent respirator under the GB 2626–2019 standard, providing filtration for nonoil-based particles comparable to United States NIOSH N95 and European FFP2 standards (22). Consistent with this classification, the standard number (GB 2626–2019), manufacturer identity, and filtration rating (KN95) are printed directly on the mask, while the manufacturing date, batch or lot number, and expiration date are printed on the product packaging, with each mask individually packaged. The respirator features an ergonomic three-dimensional design measuring 16 cm in width and 7 cm in height. The specific brand name is withheld as an institutional risk-management decision common in independent, non-sponsored comparative product evaluations. The manufacturer offers this model in a single size only, and no alternative sizes were available for selection in this study. For the active-flow FFRs, the same respirator model was used in conjunction with a portable air purification system.

**Figure 1:**
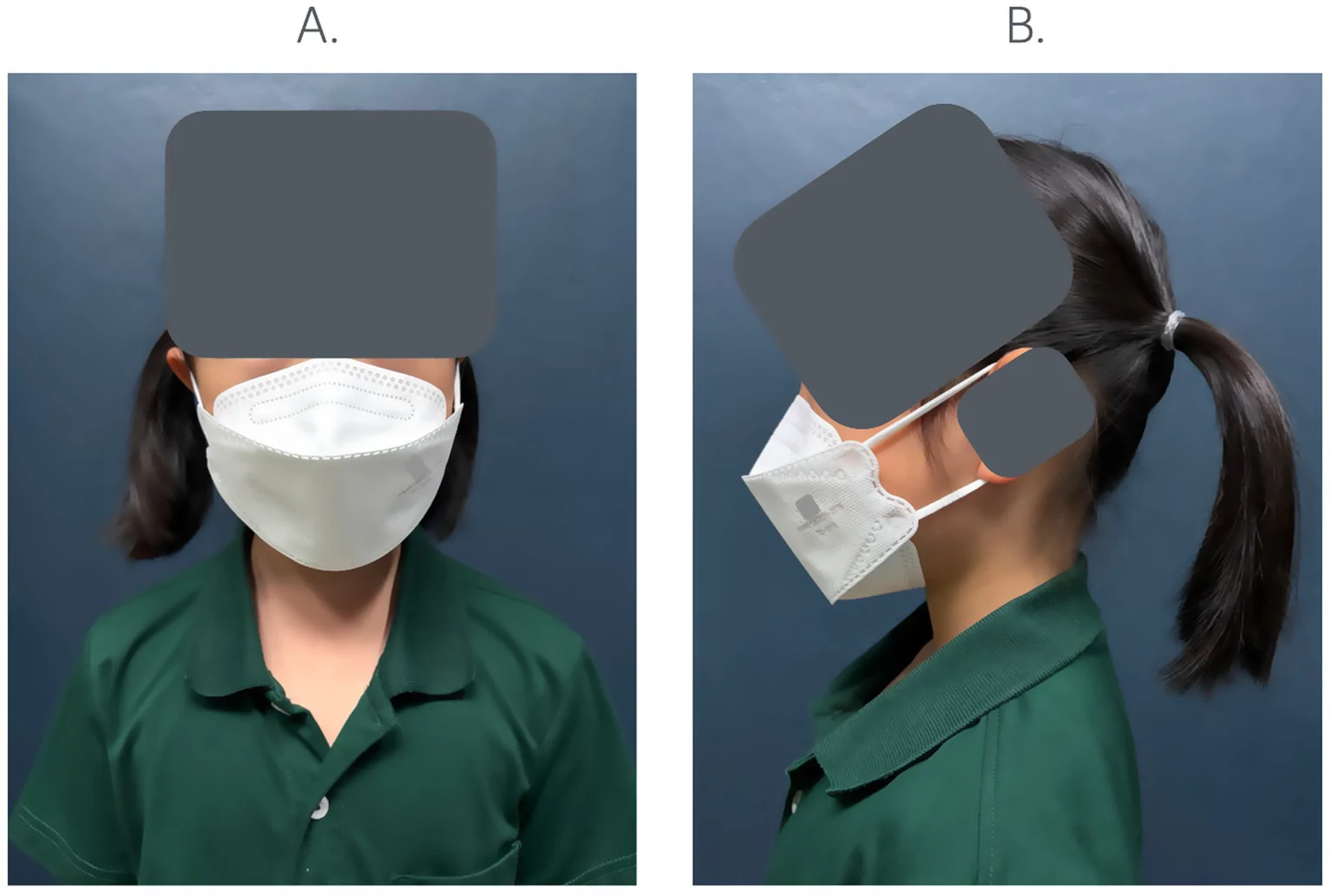
A horizontal triple-fold KN95-equivalent respirator: **(A)** anterior view and **(B)** lateral view.

### 2.3 Air purification system

The portable air purification unit weighed about 180 grams. It utilized high-efficiency particulate air (HEPA) H13-grade filtration. The device incorporated battery power and an inhalation-valve-only design consistent with a powered air-purifying respirator (PAPR), delivering an airflow volume of 66.5 L/min. The specific brand identity of the device is withheld as an institutional risk-management decision common in independent, non-sponsored comparative product evaluations. An air delivery tube connected the purification unit to an external joint port on the respirator, positioned 2 cm lateral to the midline. The tube was secured via a thin plastic ring locked from the internal aspect of the mask. Although the device is designed by the manufacturer to be worn via a neck lanyard during normal use, children in this study were instead instructed to hold the purification unit manually, keeping the tube slightly bent (Figure 2), to prevent the weight of the device from exerting tension on the tube and potentially disrupting the mask-to-face interface.

**Figure 2:**
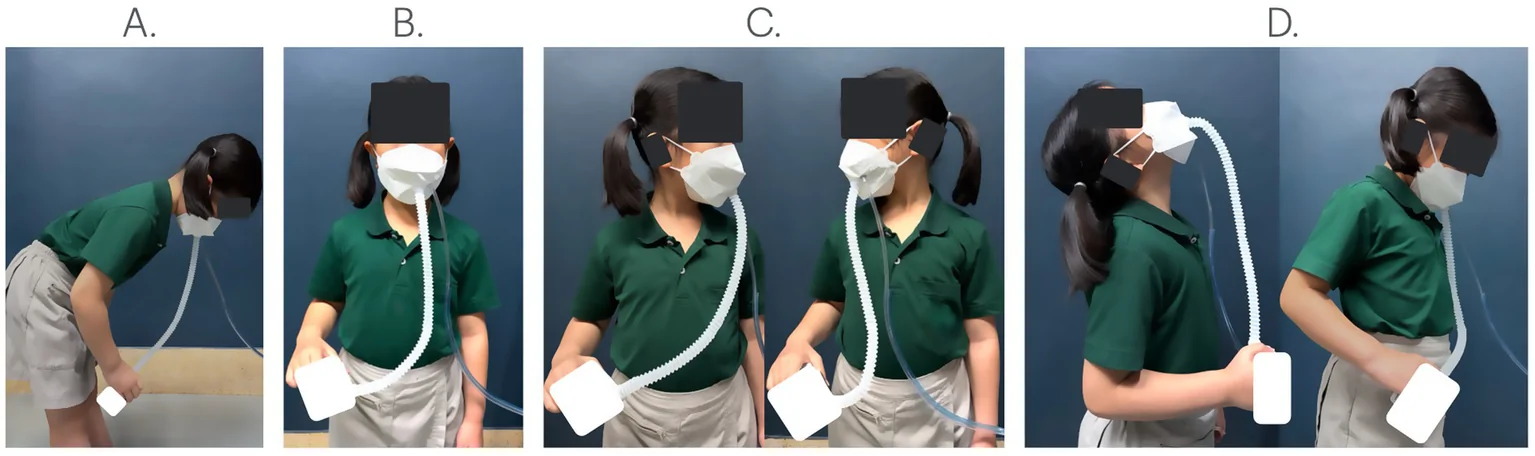
The four standardized fit-test exercises performed by a participant: **(A)** bending, **(B)** talking, **(C)** side-to-side head tuning, and **(D)** up-and-down head movement.

### 2.4 Quantitative fit testing protocol

According to the Occupational Safety and Health Administration (OSHA) (23) a quantitative fit factor measures the adequacy of a respirator seal for an individual user. This value is derived from the ratio of ambient room aerosols to the particles detected inside the facepiece while the wearer completes specific physical movements. All the quantitative evaluations in this study were executed with a TSI Portacount Pro Plus 8,038 machine from the USA following the modified Condensation Nuclei Counter protocol detailed in OSHA regulation 29 CFR 1910.134. A respirator achieved a passing grade if the overall harmonic means fit factor reached 100 or above. Testing occurred within a dedicated 20 square meter enclosed space maintained within a controlled range of 22–25 °C throughout data collection. Before initiating the equipment, researchers verbally explained the protocol and visually demonstrated the required exercises to ensure that the participants could accurately perform the necessary actions. Figure 2 illustrates the participant fit-testing procedure, which incorporated four standardized exercises: bending, talking, side-to-side head turning, and up-and-down head movement. During testing, the participant held the air purification unit under supervision, with the tube kept slightly bent to prevent the weight of the tube and unit from pulling down the upper border of the mask.

### 2.5 Anthropometric measurements

This study selected eight facial anthropometric dimensions that interface directly with respirator surfaces. These dimensions were established via several specific anatomical landmarks, as illustrated in Figure 3. The zygion (Zy) represents the most lateral point on the zygomatic arch, and the gonion (Go) represents the most lateral point at the angle of the mandible. The sellion (Se) is the point of deepest depression of the nasal bones at the nasal root. The pronasale (Pn) serves as the most anterior point of the nasal tip, whereas the subnasale (Sn) is the point where the nasal septum merges with the philtrum. Additionally, the menton (Mn) is the lowest point on the soft-tissue contour of the chin, and the tragion (Tg) is the superior point at the juncture of the tragus with the head. Based on these landmarks, eight facial dimensions were recorded, as shown in Figure 4. Interpupillary distance is the horizontal distance between the centers of the pupils. Nasal root breadth represents the horizontal width of the nose at the level of the sellion point. Bizygomatic breadth and bigonial breadth are the linear distances between the right and left zygion and gonion landmarks, respectively. The subnasale–sellion length is the linear distance between the subnasale and sellion points, and nose protrusion is the linear distance from the pronasale to the subnasale. Furthermore, menton–sellion length is the distance in the midsagittal plane from the sellion to the menton. Finally, face depth is defined as the horizontal distance between the tragion and the pronasale landmarks.

**Figure 3:**
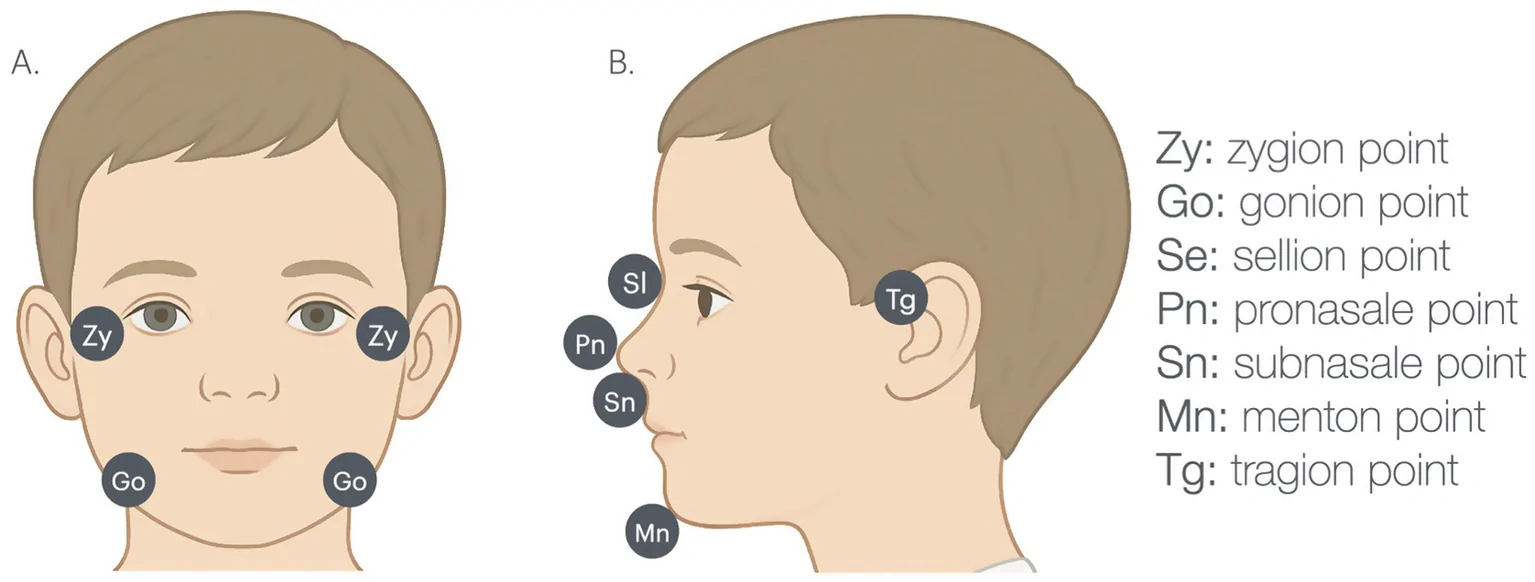
Facial landmarks: **(A)** anterior view and **(B)** lateral view.

**Figure 4:**
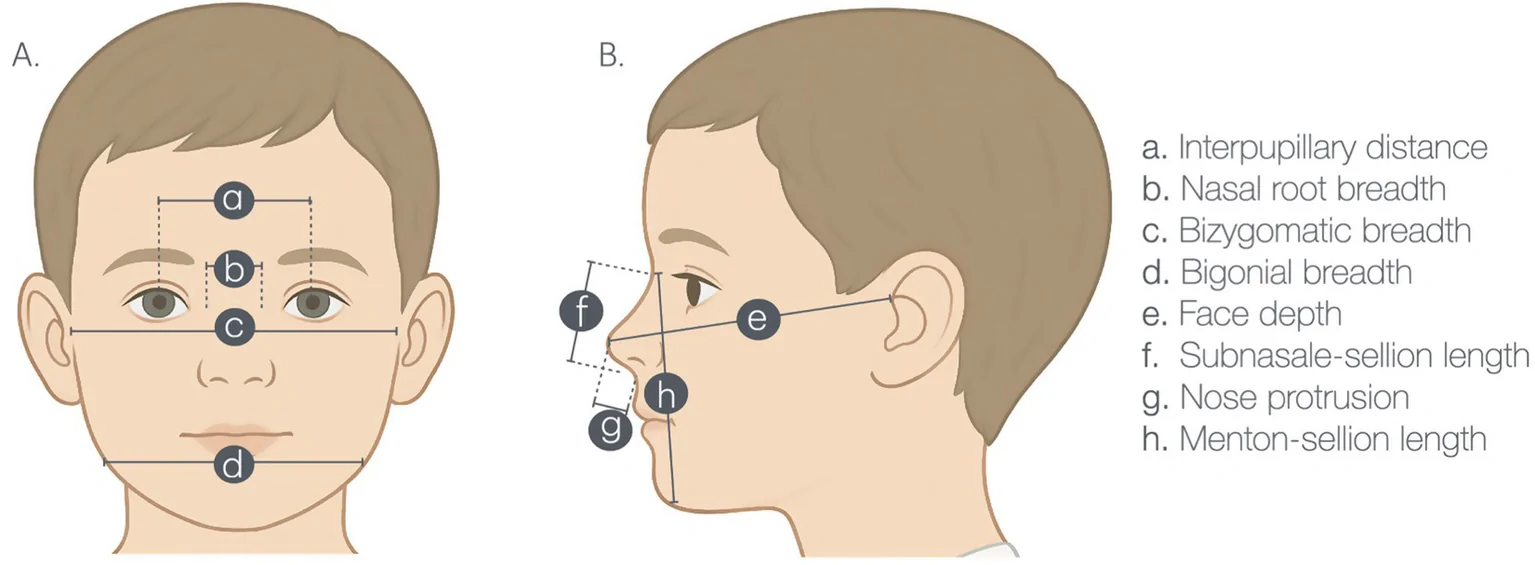
Facial dimensions: **(A)** anterior view and **(B)** lateral view.

Facial anthropometric data were collected via a 24.2 megapixel digital single-lens reflex (DSLR) camera with an 18–55 mm lens mounted on a tripod and positioned exactly one meter from each participant. Two standardized photographs were taken for every individual under natural lighting conditions to capture both frontal and lateral profiles. The camera height was strictly aligned with the eye level of the participant to ensure consistent image quality across the study. The participants were instructed to look directly into the camera lens during the frontal photograph to ensure a straight, face-on plane perpendicular to the face. For the lateral photograph, participants were asked to fix their gaze on a marker point to achieve the eye–ear plane (Frankfort horizontal plane) during image capture. To facilitate accurate identification of anatomical landmarks, researchers placed small 6 mm circular stickers directly on the zygion gonion sellion and menton points of each participant. All the acquired photographs were saved in jpeg format. These images were subsequently analyzed via Digimizer software (version 5.4.4) to digitally extract the required facial measurements.

### 2.6 Statistical analysis

Descriptive statistics are presented as arithmetic means and standard deviations for normally distributed data. The nonnormally distributed data were alternatively described as medians, together with the first and third quartiles. We used two independent sample t tests for comparisons across groups of independent, normally distributed numerical determinants and the rank-sum test for independent, nonnormally distributed numerical determinants. Comparisons across groups of categorical determinants were performed via Fisher’s exact test. Statistical estimation was covered with quantile regression for a nonnormal numerical outcome, binary logistic regression for a binary outcome, and ordinal logistic regression for an ordinal outcome. In participant demographics (Table 1), we made the comparison across independent groups of gender. Statistical tests used were independent sample t-tests for normally distributed numerical determinants and the rank-sum test for non-normally distributed numerical determinants. Comparisons across groups of categorical determinants were performed via Fisher’s exact test. In Tables 2–4, statistical estimations were covered with regression modeling accommodating paired measurements, with conditional logistic regression (Table 2), non-parametric quantile regression with clustered robust standard error correction (Table 3) and random-intercept, mixed-effect ordinal logistic regression (Table 4). Statistical tests were interpreted as statistically significant at a *p* value equal to or less than 0.05. The statistical estimation confidence interval was 95%. Missing data from one participant on facial measurements were assumed to be missing completely at random. All analyses were carried out in Stata 18.0 (College Station, TX, USA).

**Table 1:** Participant demographics, fit test pass rates, and facial dimension distributions.

Variable	Overall (*n* = 82)	Male (*n* = 47)	Female (*n* = 35)	*p* value
	Mean (SD)	Mean (SD)	Mean (SD)	
Demographic (*n* = 82)				
Age (years)	8.8 (1.3)	8.9 (1.3)	8.6 (1.3)	0.399
Height (cm)	129.8 (9.9)	130.6 (9.5)	128.7 (10.5)	0.418
Weight (kg)	29.2 (9.4)	30.3 (9.8)	27.7 (8.9)	0.228
BMI (kg/m^2^) mean (SD)	17.0 (3.8)	17.5 (4.2)	16.4 (3.2)	0.197
BMI category, *n* (%)				0.098
Underweight	5 (6.1)	4 (8.5)	1 (2.9)	
Normal	66 (80.5)	34 (72.3)	32 (91.4)	
Overweight	11 (13.4)	9 (19.1)	2 (5.7)	
The fit-test pass rate, *n* (%)				
Standard respirator	3 (3.7)	2 (4.3)	1 (2.9)	>0.999
Active-flow respirator	19 (23.2)	10 (21.3)	9 (25.7)	0.792
Facial dimension (mm) (*n* = 81)				
Anterior				
Interpupillary distance	52.1 (4.0)	52.6 (3.8)	51.6 (4.2)	0.247
Nasal root breadth	12.6 (2.0)	12.4 (2.1)	12.9 (1.9)	0.227
Bizygomatic breadth	116.0 (8.5)	117.6 (8.3)	113.9 (8.4)	0.050*
Bigonial breadth	90.3 (9.0)	91.9 (9.0)	88.1 (8.8)	0.065
Lateral				
Face depth (tragion-pronasale)	82.5 (5.6)	83.8 (5.4)	80.6 (5.6)	0.010*
Subnasale-sellion length	34.0 (2.7)	34.3 (2.9)	33.7 (2.4)	0.368
Nose protrusion	13.9 (1.7)	14.0 (1.9)	13.7 (1.5)	0.477
Menton-sellion length	80.7 (7.6)	81.1 (7.7)	80.1 (7.4)	0.568

Fit-test passing was defined as an overall harmonic mean fit factor ≥100, in accordance with the OSHA quantitative fit-testing protocol (23). Wilcoxon rank-sum test for skewed variables. Statistical significance: * for *p* ≤ 0.05, ** for *p* ≤ 0.01; BMI, body mass index.

**Table 2:** Comparison of fit-test pass rates and fit factors between standard FFRs and active-flow FFRs across different activities (*n* = 82).

Activity	Standard FFRs, median (Q1-Q3) (*n* = 82)	Active-flow FFRs, median (Q1-Q3) (*n* = 82)	Odds ratio^a^ or median difference^b^	*P* value
The fit-test pass rate, *n* (%)	3 (3.7)	19 (23.2)	17.0 (2.3, 127.8) *^a^*	0.006 ^a,^**
Fit factor				
Overall fit factor	9.5 (5.0–18.0)	39.5 (20.0–64.0)	30.0 (21.8, 38.2) *^b^*	<0.001 ^b,^**
Bending	8.5 (4.0–24.0)	58.5 (38.0–111.0)	50.0 (36.8, 63.2) *^b^*	<0.001 ^b,^**
Talking	13.5 (6.0–29.0)	59.5 (41.0–94.0)	47.0 (36.1, 57.9) *^b^*	<0.001 ^b,^**
Side-to-side head turning	7.5 (4.0–14.0)	49.0 (18.0–73.0)	42.0 (29.0, 55.1) *^b^*	<0.001 ^b,^**
Up-and-down head movement	8.0 (4.0–15.0)	35.5 (11.0–81.0)	28.0 (14.3, 41.7) *^b^*	<0.001 ^b,^**

Fit test pass rates were analyzed via conditional logistic regression to account for the pair-matched nature of the data. The five dimensions of the fit factor were compared via nonparametric quantile regression to estimate median differences, and clustered robust standard errors were used to account for the pair-matched data. ^a^Estimated via conditional logistic regression; ^b^Derived via quantile regression; Statistical significance: ** for *p* ≤ 0.01; FFRs, filtering facepiece respirators.

**Table 3:** Associations between facial dimensions and fit test pass rates for the active-flow FFRs (*n* = 19).

Facial dimension	Crude odds ratios (95% CI)	*p* value	Adjusted odds ratios (95% CI)	*p* value
Anterior				
Interpupillary distance	0.99 (0.88, 1.11)	0.842	0.67 (0.49, 0.91)	0.009**
Nasal root breadth	0.88 (0.67, 1.14)	0.324	0.85 (0.65, 1.11)	0.233
Bizymatic breadth	1.07 (1.00, 1.14)	0.052	1.31 (1.10, 1.56)	0.002**
Bigonial breadth	1.06 (1.00, 1.12)	0.056	0.94 (0.83, 1.06)	0.287
Lateral				
Face depth (tragion -pronasale)	1.04 (0.95, 1.13)	0.404	0.96 (0.79, 1.17)	0.687
Subnasale-sellion length	1.02 (0.86, 1.22)	0.814	1.01 (0.69, 1.49)	0.957
Nose protrusion	1.06 (0.77, 1.44)	0.737	1.01 (0.65, 1.55)	0.977
Menton-sellion length	1.07 (1.01, 1.14)	0.020	1.11 (1.02, 1.22)	0.018*

All analyses were based on the active-flow FFR pass rate. Crude odds ratios (cORs) were determined via univariable binary logistic regression to assess the association of each facial dimension independently. Adjusted odds ratios (aORs) were estimated via multivariable binary logistic regression that included all eight facial dimensions simultaneously; crude odds ratio indicated unadjusted magnitude of association for each facial measurement separately; adjusted odds ratio reflected magnitude of each facial measurement when the other seven facial measurements were considered roughly equal. Statistical significance: * for *p* < 0.05; ** for *p* < 0.01.

**Table 4:** Comparison of perceptions between standard and active-flow FFRs (*n* = 82).

Parameter (1–3 scale)	Standard FFRs, n (%) (*n* = 82)	Active-flow FFRs, *n* (%) (*n* = 82)	Proportional odds ratio*^a^*	*P* value
Comfort: easy breathing				
Difficult	3 (3.7%)	1 (1.2%)	10.31 (4.34, 24.55)	<0.001**
Neutral	48 (58.5%)	10 (12.2%)		
Easy	31 (37.8%)	71 (86.6%)		
Comfort: feeling unfit				
Fit	7 (8.5%)	9 (11.0%)	0.75 (0.51, 1.10)	0.142
Neutral	31 (37.8%)	35 (42.7%)		
Loose	44 (53.7%)	38 (46.3%)		
Comfort: irritating to the skin				
Least comfortable	2 (2.4%)	2 (2.4%)	1.00 (0.65, 1.54)	>0.999
Neutral	21 (25.6%)	21 (25.6%)		
Most comfortable	59 (72.0%)	59 (72.0%)		
Comfort: inconvenience				
Convenient	13 (15.9%)	2 (2.4%)	6.17 (3.07, 12.42)	<0.001**
Neutral	40 (48.8%)	17 (20.7%)		
Inconvenient	29 (35.4%)	63 (76.8%)		

Perceptions of respirator use, measured on a 3-point Likert scale, were analyzed via univariable, mixed-effects proportional odds ordinal logistic regression to evaluate shifts toward higher categories when the respirator was augmented with active-flow positive pressure. ^a^Estimated from the proportional odds ordinal logistic regression; Statistical significance: ** for *p* ≤ 0.01; FFRs, filtering facepiece respirators.

## 3 Results

### 3.1 Participant characteristics and fit pass rate

The participant demographics, fit test pass rates, and facial dimension distributions are presented in Table 1. A total of 82 children, consisting of 47 males (57.3%) and 35 females (42.7%), were included in the analysis. The mean age was 8.8 years, with a standard deviation of 1.3 years, and there were no significant differences between males and females in terms of age (*p* = 0.399), height (*p* = 0.418), weight (*p* = 0.228), or body mass index (BMI) (*p* = 0.197). Most participants were within the normal BMI range (80.5%), while 6.1% were underweight and 13.4% were overweight. The overall passing rate was 3.7% (3 of 82) under the standard FFRs, with a median (Q1–Q3) overall fit factor of 151.0 (133.0–189.0) in this group. This increased to 23.2% (19 of 82) when positive pressure was applied, with a median (Q1–Q3) overall fit factor of 127.0 (111.0–145.0) in this group. Males had slightly larger facial dimensions, and two parameters showed statistically significant differences. Bizygomatic breadth was greater in males (*p* = 0.050), and face depth was greater in males (*p* = 0.010). Interpupillary distance, nasal root breadth, bigonial breadth, subnasale-sellion length, nose protrusion, and menton-sellion length were not significantly different.

### 3.2 Impact of respirator type and exercise on the median fit factor

The median fit factors for overall performance and specific exercises, including bending, talking, turning head side to side, and moving head up and down, were compared by gender and are presented in Figure 5. For standard FFR use, the overall median fit factor was 9.0 for males and 10.0 for females. The highest median fit factor occurred during talking, with values of 13.0 for males and 14.0 for females. The median fit factors for bending, turning head side to side, and moving head up and down were 10 or less for both gender groups. Use of the active-flow FFR increased fit factors across all activities. The overall median fit factor rose to 42.0 for males and 35.0 for females. For males, the median fit factor exceeded 50 during bending (58.0), talking (57.0), and turning head side to side (53.0), while moving head up and down remained lower at 38.0. For females, two activities exceeded a median fit factor of 50, bending (59.0) and talking (69.0). Turning head side to side and moving head up and down had median fit factors of 41.0 and 33.0, respectively. No statistically significant differences were observed between males and females for any individual exercise or for the overall fit factor when the standard FFRs were compared against the active-flow FFRs (Supplementary Table S1).

**Figure 5:**
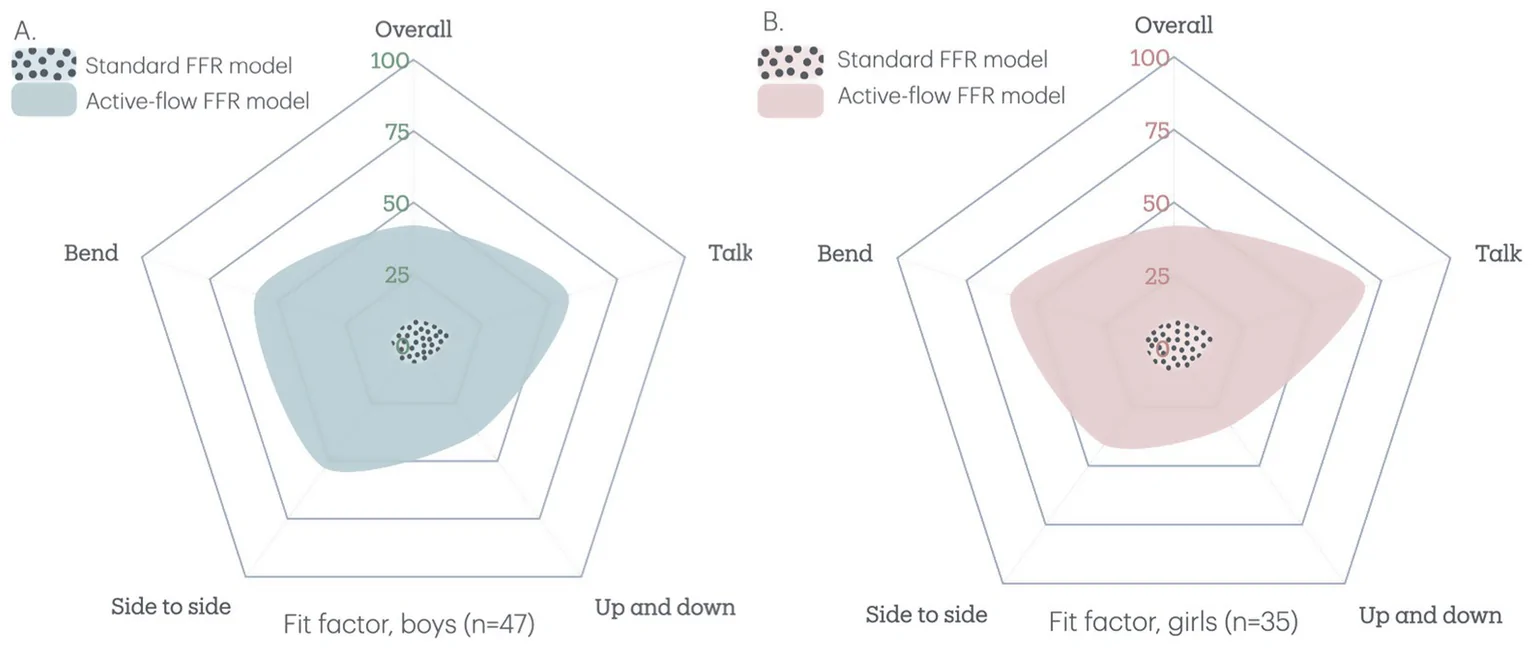
Median fit factors for standard and active-flow filtering facepiece respirators (FFR): **(A)** male and **(B)** female.

Table 2 presents a comparison of fit test performance between standard and active-flow FFRs among all participants. The overall fit test pass rate increased from 3.7% with the standard respirator to 23.2% with the active-flow respirator, with an odds ratio of 17.0 (95% CI 2.3–127.8; *p* = 0.006). Both the overall comparison and all activity-specific comparisons demonstrated statistically significant improvements with the active-flow respirator (*p* < 0.001). The overall median fit factor increased from 9.5 (Q1–Q3, 5.0–18.0) to 39.5 (Q1–Q3, 20.0–64.0), with a median difference of 30.0 (95% CI 21.8–38.2; *p* < 0.001). During bending, the median fit factor increased from 8.5 (Q1–Q3, 4.0–24.0) to 58.5 (Q1–Q3, 38.0–111.0), with a median difference of 50.0 (95% CI 36.8–63.2; *p* < 0.001). During the talking exercise, the median fit factor increased from 13.5 (Q1–Q3, 6.0–29.0) to 59.5 (Q1–Q3, 41.0–94.0), with a median difference of 47.0 (95% CI 36.1–57.9; *p* < 0.001). For side-to-side head turning, the median fit factor increased from 7.5 (Q1–Q3, 4.0–14.0) to 49.0 (Q1–Q3, 18.0–73.0), with a median difference of 42.0 (95% CI 29.0–55.1; *p* < 0.001). Finally, for up-and-down head movement, the median fit factor increased from 8.0 (Q1–Q3, 4.0–15.0) to 35.5 (Q1–Q3, 11.0–81.0), with a median difference of 28.0 (95% CI 14.3–41.7; *p* < 0.001).

### 3.3 Comparative fit performance and facial dimensions

In the positive pressure condition, specific facial dimensions were significantly associated with fit-test success (Table 3). An increased interpupillary distance was associated with lower odds of passing, with an adjusted odds ratio (aOR) of 0.67 (95% CI 0.49–0.91; *p* = 0.009). Conversely, dimensions associated with higher odds of passing were identified in both anterior and lateral measurements. With respect to anterior dimensions, greater bizygomatic breadth was associated with increased odds of passing (aOR 1.31; 95% CI 1.10–1.56; *p* = 0.002). For lateral dimensions, menton–sellion length was also associated with increased odds of success (aOR 1.11; 95% CI 1.02–1.22; *p* = 0.018). Other facial dimensions, including nasal root breadth, bigonial breadth, face depth, subnasale–sellion length, and nose protrusion, did not demonstrate statistically significant associations in either the crude or adjusted models.

### 3.4 Comparison of comfort perceptions between respirator types

Comparisons of physiological and comfort perceptions between the standard and active-flow FFRs are presented in Table 4. Participants reported easy breathing significantly more frequently with the active-flow respirator (86.6%) than with the standard respirator (37.8%), with a proportional odds ratio of 10.31 (95% CI 4.34–24.55; *p* < 0.001). However, inconvenience was also reported more frequently with the active-flow respirator (76.8%) than with the standard respirator (35.4%), with a proportional odds ratio of 6.17 (95% CI 3.07–12.42; *p* < 0.001). Perceptions regarding fit (feeling unfit) and skin irritation did not differ significantly between the two respirator conditions.

## 4 Discussion

### 4.1 Facial anthropometry

In this study, we evaluated 82 children between the ages of 5 and 10 years to compare the performance of standard FFRs and active-flow disposable FFRs. We specifically assessed the correlation between mask fit and facial dimensions via a 2D photogrammetric methodology consistent with protocols previously applied to adult cohorts (24). This method was chosen because 3D scanning equipment was unavailable, and direct caliper-based anthropometry demands a level of sustained positional cooperation that is difficult to achieve accurately in this population. The reliability of 2D photogrammetry relative to direct measurement has previously been validated for respirator-relevant facial dimensions. A validation study assessing ten of these dimensions found that seven of the eight dimensions used in the present study, namely bizygomatic breadth, bigonial breadth, menton–sellion length, interpupillary distance, nose protrusion, nasal root breadth, and subnasale–sellion length, achieved good-to-excellent intraclass correlation coefficients of 0.81 or higher (25). Face depth was not among the dimensions evaluated in that validation study, and its findings in the present study should therefore be interpreted with appropriate caution. This approach differs from previous studies in children that utilized manual calipers (26, 27) or 3D scanning (28, 29) to assess facial anthropometrics. Although differences in measurement techniques across studies prevent direct comparisons, the data suggest a trend toward smaller facial dimensions in our study population. For example, 3D anthropometric data from a Latvian cohort of 5-year-olds revealed bizygomatic breadths of 116.7 ± 3.8 mm in males and 112.6 ± 3.7 mm in females (26). These figures are consistent with our observed means for the broader 5- to 10-year-old age group, which were 117.6 ± 8.3 mm in males and 113.9 ± 8.4 mm in females. Furthermore, research in a Romanian cohort utilizing manual techniques reported bizygomatic and bigonial widths of 116.9 mm and 93.5 mm for a younger age group of 5–6.5 years (27). These values are remarkably close to our observed means of 116.0 ± 8.5 mm and 90.3 ± 9.0 mm. The current findings demonstrate that males have significantly larger midfacial measurements, particularly with respect to bizygomatic breadth and face depth. This observation is consistent with previous research involving children. Although traditional perspectives suggest that pronounced gender dimorphism in facial structure is primarily a postpubertal phenomenon (30, 31), the present results support recent evidence that these morphological changes begin much earlier in childhood (14, 15). These findings indicate that gender differences necessitate careful consideration during facial interventions in children. Furthermore, the development of population-specific databases may be highly beneficial for clinical practice and precision medicine.

### 4.2 Fit pass rates

The major findings of this research indicate that the standard FFR reached a pass rate of only 3.7% among participants. The median fit factor was 9 for males and 10 for females. These values fail to meet the established passing standard of 100. The low passing rate indicates that children may not receive adequate protection from ambient air pollutants when these devices are used. These findings align with earlier adult research regarding ear-loop masks. One study reported that surgical and shell masks achieved a mean fit factor of only 3.2 (32). Furthermore, an evaluation of nine CE-marked ear-loop respirator models revealed that only two of ninety tests reached an acceptable fit factor (13). When considering only the children who passed under standard respiratory conditions, the fittest three children had a median overall fit factor of 151.0, compared to a median of 127.0 among the 19 participants who passed under active-flow FFRs. This lower median in the active-flow group likely reflects a difference in group composition. The three children who passed under standard conditions already had a well-fitting mask, whereas most of the 19 children who passed only after active-flow was applied did not fit the mask as well to begin with. Active-flow was enough to raise their fit factor above the passing threshold, but not to the same degree. However, when all 82 participants are considered together, active-flow improved the overall fit factor, with median fit factors under active-flow conditions of 42 for males and 35 for females. Overall, the passing rate increased significantly from 3.7% under standard conditions to 23.2% when the FFR was modified to provide a continuous flow of clean air (OR = 17.0, *p* < 0.006). Although the portable air purifier used in this study generated an airflow of 66.5 L/min, which falls below the NIOSH certification standard of 170 L/min for loose-fitting powered air-purifying respirators (21), a significant improvement in performance was nonetheless achieved. Owing to the current paucity of research specifically examining the efficacy of loose-fitting PAPRs for mitigating environmental pollution exposure (7) it remains difficult to elucidate why an airflow lower than the standard recommendation still yields significant benefits. In reviewing previous literature sharing a similar conceptual framework, a pilot study focused on healthcare cleaning services performing moderate workload activities reported that the minute volume, mean inhalation flow, and average peak inhalation flow rates were 41, 97, and 145 L/min, respectively (33). These values are all notably lower than the 170 L/min minimum required for NIOSH-approved devices (21). Such findings suggest that the 170 L/min threshold was likely established for high-exertion industrial environments and may exceed the physiological breathing demands of participants in light activity settings. The effectiveness observed at 66.5 L/min could be attributed to the light activity level of the participants during the protocol. To enhance these outcomes and ensure adequate protection during diverse real-life scenarios, future research should focus on determining the minimum operational flow requirements specifically for children across various activity levels. Additionally, investigating the improvement of pass rates via portable high-volume units capable of matching airflow to physical exertion would be beneficial for developing population-specific respiratory protection.

### 4.3 Movements and leakage

Beyond respirator design specifications, the activation of facial muscles during head movement or expression significantly alters the contact surface between the mask and the face. These physiological factors directly contribute to increased leakage and a subsequent reduction in protective efficacy, which varies in degree among individual users (34–36). The association between specific head movements and protection efficiency appears to vary depending on the study setting and protocol. Previous research in adults reported that, among FFP3 respirators, trunk flexion or bending yielded a lower fit factor than talking or moving the head in side-to-side and up-and-down directions (32). Conversely, research involving Thai healthcare personnel indicated that N95 respirators with a three-panel flat-fold design recorded the lowest fit values during head-up and-down movements compared with talking and side-to-side head movements (36). Continuous airflow respirators are intended to maintain a positive pressure environment and fill the gap between the facial surface and the respirator. Although these systems significantly improved the overall fit factor across all exercises, the impact of postural variations remained significant. During bending, talking, and side-to-side movements, the odds ratios ranged between 42 and 50. In contrast, moving the head up and down yielded the lowest odds ratio of 28.

The specific physiological mechanisms for this variation within the current protocol remain unclear; however, the data suggest that certain facial dimensions may be positively associated with passing the fit test following the application of continuous clean air. Specifically, a potential positive correlation was observed with bizygomatic breadth and menton-sellion length. These findings might indicate that a larger facial surface area allows for a more stable overall interface with the respirator. Conversely, a negative association passing the fit test was observed only for the interpupillary distance. It has been reported that, across populations, greater interorbital width and intercanthal distance tend to show a moderate correlation with a wider nasal bridge (37). These findings suggest that a wider distance between the eyes may inadvertently hinder the effectiveness of the nose clip seal. Consequently, individuals with a greater interpupillary distance may exhibit increased susceptibility to leakage in the nasal region during vertical head movements. This observation aligns with prior research utilizing head form simulations, which identified the nasal bridge as the primary site of leakage (38). Furthermore, evaluations of various ear loop mask designs have confirmed that these respirators consistently demonstrate leakage around the nose seal (13). The current findings suggest that although continuous airflow significantly enhances mask efficacy, the extent of this improvement is modulated by both postural variations and individual facial dimensions. These results indicate that future investigations should prioritize these specific leakage pathways to enhance the structural integrity and seal the upper mask border.

### 4.4 Safety and acceptability

User safety and acceptability are essential factors in mask design, particularly for children (39–42). Clinical evaluations of these issues in children remain limited, although the prevailing trend suggests that wearing face masks does not result in profound negative safety impacts or adverse perceptions. For example, a study of 20 children aged 8–10 years wearing FFP2 or N95 masks during exercise revealed no evidence of hypercapnia or hypoxia (42). Similarly, a study of 38 children aged 8–12 years reported no clinically relevant changes in cardiorespiratory parameters or perceptions of exertion when surgical masks were used (41). These findings are further supported by a randomized crossover trial of 106 children, which demonstrated that N95 masks equipped with microfans are safe for use in children, as carbon dioxide values remain within safe physiological thresholds (39). Direct physiological parameters were not measured. Instead, perceptions were captured using a visual analog scale. Although the active flow condition significantly enhanced breathing, it also led to a higher perception of inconvenience regarding the prototype. It is therefore critical to address these ergonomic concerns during the design phase. The development of a respirator that combines high clinical effectiveness with acceptance and compliance among children represents a significant research gap that must be addressed in future iterations.

## 5 Strengths and limitations

A primary strength of this study is the objective examination of mask performance in children using the gold-standard TSI Portacount Pro Plus 8,038. This quantitative method provides reliable data on how masks protect children while exploring affordable active-flow modifications for diverse facial shapes. However, several limitations should be noted. The study was restricted to a single mask model, which may limit the generalizability of our findings to other designs. Additionally, 2D measurements may not fully capture the 3D complexities of facial contours, and the brief testing period may not reflect the effects of long-term use. Finally, because the air purifier was originally designed for adults, its weight required participants to hold the unit manually to prevent it from compromising the mask seal. This manual support may have provided a degree of external stabilization not achievable during unsupervised, routine use, and could have artificially enhanced the face seal. The reported fit and performance results should therefore be interpreted as reflecting a supervised, best-case testing condition rather than real-world daily wear.

## 6 Conclusion

This evaluation indicates that disposable, horizontal triple-fold KN95 respirators provide very limited protection for children aged five to ten, yielding a passing rate of only 3.7%. While active flow systems increased the passing rate to 23.3%, protection levels remained significantly below established safety thresholds. Reduced filtration efficiency was closely correlated with wider interpupillary distances and specific physical activities, such as vertical head movements. On the basis of these findings, commonly available consumer-market respirators are currently unsuitable as a primary prevention strategy. Policymakers and healthcare providers should prioritize evidence-based data when advising families on air pollution mitigation for children. Future research should focus on refining the upper-edge seal and developing portable units that provide sufficient airflow during real-world activity while enhancing user convenience to better accommodate the physiological needs of children.

## Data Availability

The raw data supporting the conclusions of this article will be made available by the authors, without undue reservation.
