# Supplementary material for: Active-flow systems enhance respirator fit success in children: a comparative study with standard disposable models
Source: Front Public Health. 2026 Oct 14;14:1851331. doi: 10.3389/fpubh.2026.1851331 (PMC13714880; doi:10.3389/fpubh.2026.1851331)
Supplement: Supplementary file 1 [file Table_1.docx]

**Table S1.** Fit factors by gender across exercises for standard and active-flow FFRs (N = 82).

| **Activity** | **Standard FFRs** | | | **Active-Flow FFRs** | | |
| --- | --- | --- | --- | --- | --- | --- |
|  | **Male (n = 47)** | **Female (n = 35)** | ***p* value** | **Male (n = 47)** | **Female (n = 35)** | ***p* value** |
| Overall fit factor | 9.0 (4.0–18.0) | 10.0 (5.0–18.0) | 0.46 | 42.0 (16.0–64.0) | 35.0 (22.0–101.0) | 1.00 |
| Bending | 8.0 (4.0–20.0) | 10.0 (5.0–36.0) | 0.22 | 58.0 (41.0–100.0) | 59.0 (36.0–153.0) | 0.69 |
| Talking | 13.0 (5.0–22.0) | 14.0 (7.0–42.0) | 0.26 | 57.0 (41.0–86.0) | 69.0 (41.0–102.0) | 0.43 |
| Side-to-side head turning | 7.0 (5.0–13.0) | 8.0 (4.0–22.0) | 0.68 | 53.0 (15.0–73.0) | 41.0 (21.0–86.0) | 0.73 |
| Up-and-down head movement | 7.0 (4.0–14.0) | 8.0 (4.0–17.0) | 0.48 | 38.0 (8.0–80.0) | 33.0 (13.0–128.0) | 0.40 |

*Values are median (Q1–Q3) fit factor. Fit factors were compared between female and male participants within each respirator condition using the rank-sum test for independent groups. Abbreviations: FFRs, filtering facepiece respirators.*
